# Left Shoulder Cystic Lesion Mimicking a Soft Tissue Mass: A Rare Presentation of the Geyser Phenomenon

**DOI:** 10.7759/cureus.111356

**Published:** 2026-06-23

**Authors:** Mohammed Mushabbab Alobud, Raghad Mahdi M Al-Awn, Mahmoud Rezk Hussein, Mohammed A AlOmran, Ghazi M Albarqi, Sayed Agha Ali Shah

**Affiliations:** 1 Orthopedic Surgery, Armed Forces Hospital - Southern Region, Khamis Mushait, SAU; 2 General Practice, Ahad Rufaidah General Hospital, Ahad Rufaidah, SAU; 3 Histopathology, Armed Forces Hospital - Southern Region, Khamis Mushait, SAU; 4 Orthopedics, Armed Forces Hospital - Southern Region, Khamis Mushait, SAU

**Keywords:** acromioclavicular joint cyst, geyser phenomenon, rotator cuff tears, shoulder pseudocyst, soft tissue mass

## Abstract

The geyser phenomenon is a rare complication of chronic rotator cuff disease in which synovial fluid passes from the glenohumeral joint through a rotator cuff defect into the acromioclavicular (AC) joint, resulting in a cystic swelling over the shoulder. Because these lesions can enlarge over time and exhibit atypical imaging features, they may be mistaken for soft tissue tumors. We report the case of a 78-year-old Saudi man with diabetes mellitus, hypertension, and rheumatoid disease who presented with a slowly enlarging left shoulder mass that had been present for 1.5 years. Magnetic resonance imaging (MRI) demonstrated a 5.7-cm complex cyst communicating with the AC joint, associated with massive full-thickness rotator cuff tears and advanced degenerative changes. Follow-up imaging showed interval enlargement of the lesion, hemorrhagic contents, and a new lytic lesion in the lateral clavicle, raising concern for infection or malignancy. The patient underwent surgical excision of the lesion with distal clavicle resection. Histopathological examination revealed a benign inflammatory pseudocyst with no evidence of malignancy. This case illustrates an unusual presentation of the geyser phenomenon that clinically and radiologically mimicked a soft tissue neoplasm. Awareness of this entity may help avoid unnecessary investigations and facilitate the establishment of the correct diagnosis.

## Introduction

An acromioclavicular (AC) joint cyst is a rare condition that typically develops secondary to chronic rotator cuff pathology and consists of thick, viscous fluid enclosed within a fibrous capsule [1]. AC joint cysts are classified as type I or type II based on their underlying etiology [2]. The geyser phenomenon is an uncommon imaging finding characterized by synovial fluid escaping through a rotator cuff defect, traversing the subacromial bursa, and extending through a degenerated AC joint, resulting in capsular distension and cyst formation [3]. Because these lesions may present as progressively enlarging masses with atypical imaging features, they are frequently mistaken for soft tissue neoplasms. We report a case of the geyser phenomenon in an elderly man with multiple comorbidities and a family history of malignancy who presented with a large shoulder mass that initially raised concern for a soft tissue tumor. This case highlights the importance of accurate clinical, radiological, and histopathological evaluation in establishing the correct diagnosis and guiding appropriate management.

## Case presentation

A 78-year-old Saudi man presented to the orthopedic clinic with a swelling over his left shoulder that had been present for approximately 1.5 years. The mass was initially small but progressively enlarged, as demonstrated on magnetic resonance imaging (MRI) studies performed in October 2025 and February 2026. He reported no pain, skin changes, ulceration, constitutional symptoms, or restriction of shoulder movement, and remained able to perform his usual daily activities without difficulty. His medical history was significant for type 2 diabetes mellitus, with a recent hemoglobin A1c level of 7.92%, hypertension, and rheumatoid disease associated with neuropathy causing numbness for six years. He also had a history of three cardiac catheterizations, the most recent having been performed one year prior to presentation. The patient reported a previous traumatic shoulder injury involving dislocation and ligament tear. He was a non-smoker and had previously served in the military. His family history was notable for a cousin who underwent amputation for a joint tumor that was subsequently diagnosed as malignant.

Physical examination

**Figure 1:**
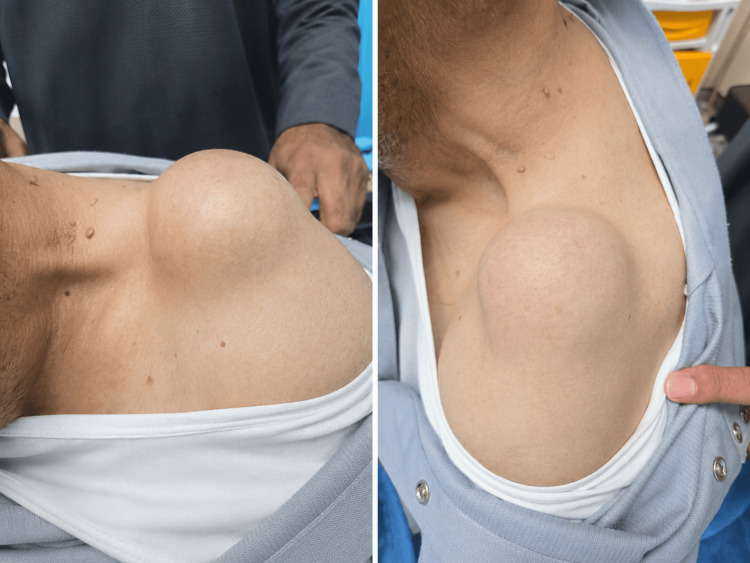
Clinical photographs demonstrating a large, smooth, well-circumscribed swelling over the superior aspect of the shoulder and acromioclavicular region, producing a prominent dome-shaped mass without overlying skin discoloration or ulceration. The lesion is consistent with a giant acromioclavicular joint cyst (Geyser phenomenon), associated with chronic rotator cuff pathology.

During physical examination, the patient was conscious, oriented, and not in distress, with stable vital signs and no evidence of pallor, cyanosis, or lymphadenopathy. On inspection, there was a visible oval swelling over the superior aspect of the left AC joint region, measuring approximately 7 × 4.5 × 4 cm. The overlying skin was intact, with no erythema, ulceration, dilated veins, sinus formation, or discharge. On palpation, the swelling was normothermic and non-tender to mildly tender, with a cystic to firm consistency, a smooth surface, and well-defined margins. It was slightly mobile but appeared attached to deeper structures, likely the AC joint. Fluctuation was positive, transillumination was negative, and the swelling was non-pulsatile. The range of motion of the shoulder was full and painless. On special testing, cross-body adduction was mildly positive, whereas other impingement tests were unremarkable. Neurovascular examination was normal, with intact distal pulses, sensation, and motor function (Figure 1).

Laboratory investigations

Routine laboratory investigations revealed metabolic abnormalities, including elevated fasting glucose (8.02 mmol/L (144.36 mg/dL)) and blood urea nitrogen (7.80 mmol/L (21.8 mg/dL)). Alkaline phosphatase was 98 U/L and within the normal reference range. The lipid profile demonstrated dyslipidemia, with elevated triglycerides (2.73 mmol/L (242 mg/dL)) and decreased high-density lipoprotein (HDL) cholesterol (0.94 mmol/L (36 mg/dL)). Hematological parameters were within normal limits, with a white blood cell count of 7.70 × 10⁹/L, hemoglobin level of 15.20 g/dL, and platelet count of 193 × 10⁹/L. Additional investigations showed an elevated HbA1c level of 7.92%, whereas thyroid-stimulating hormone (TSH) was 2.43 µIU/mL, vitamin B12 was 513 pg/mL, and corrected calcium was 2.37 mmol/L, all within normal reference ranges.

Radiological findings

**Figure 2:**
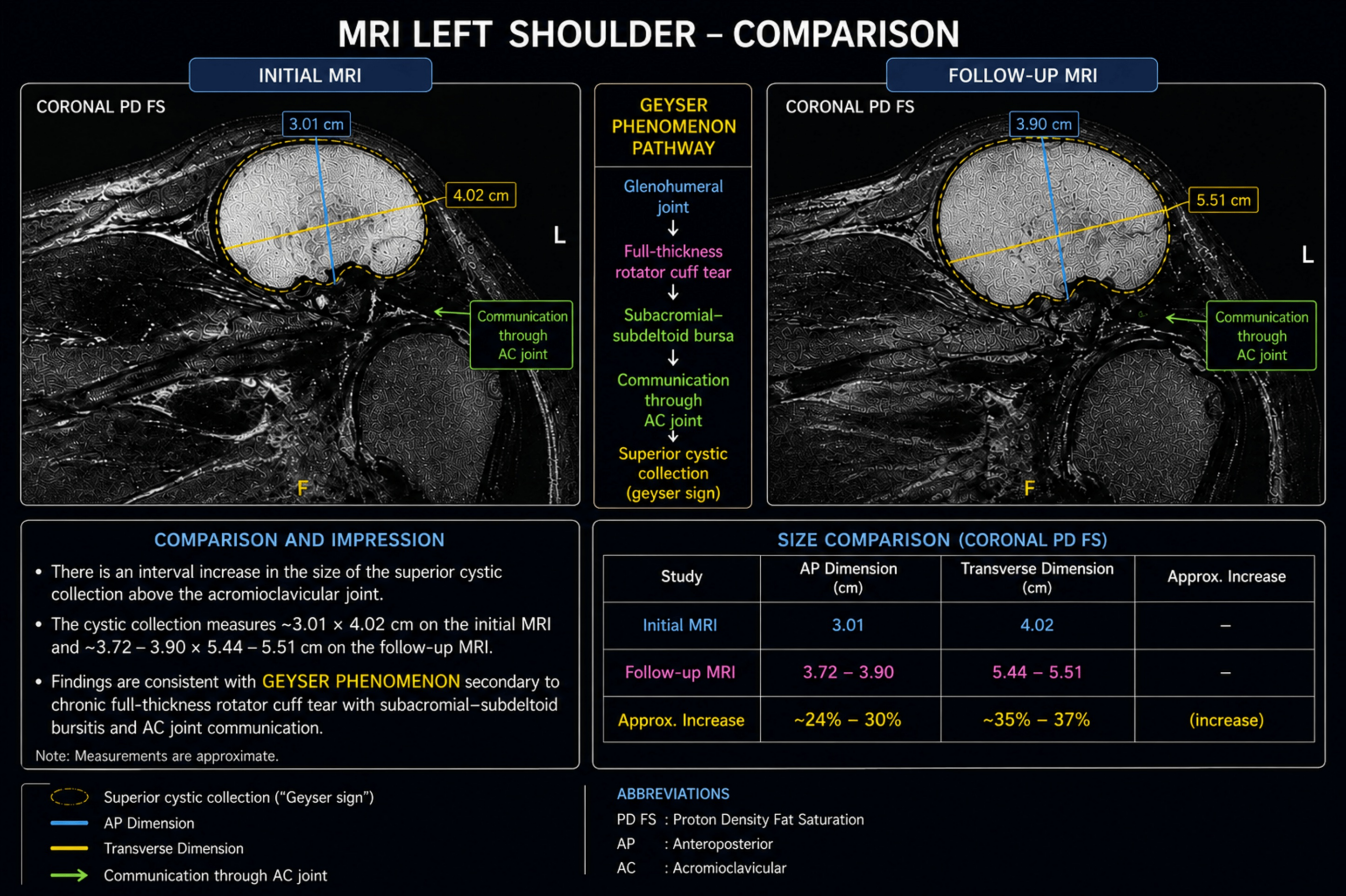
Comparison of the serial MRI of the current patient, demonstrating progressive enlargement of the acromioclavicular (AC) joint cyst (Geyser phenomenon). Initial and follow-up MRI examinations of the patient's left shoulder demonstrated interval enlargement of the cystic lesion, with persistent communication with the acromioclavicular joint. Note: The annotations, labels, and layout were created by the authors using Canva® (Canva Pty Ltd., Sydney, Australia) based on the patient’s original MRI images. No AI-generated images were used.

**Figure 3:**
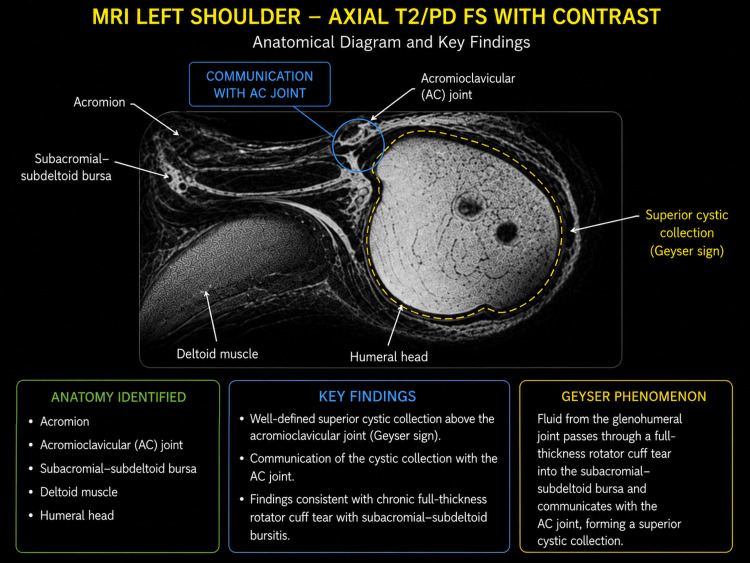
Axial MRI image of the current patient, demonstrating a cystic lesion communicating with the acromioclavicular (AC) joint, consistent with the Geyser phenomenon. Arrows indicate the communication pathway between the cyst and the acromioclavicular joint. Note: Annotations were added by the authors using Canva® (Canva Pty Ltd., Sydney, Australia) based on the patient’s original MRI image. No AI-generated images were used.

MRI studies performed several months apart demonstrated marked degenerative changes of the AC joint, along with a well-defined globular cystic lesion overlying the joint, measuring approximately 3.3 × 4.4 cm and showing predominantly fluid signal intensity with mixed intraluminal components. There were also full-thickness tears of the supraspinatus, infraspinatus (with tendon retraction and muscle atrophy), and subscapularis tendons. The long head of the biceps tendon was not visualized, likely due to rupture. In addition, superior subluxation of the humeral head was noted, along with moderate joint effusion and possible intra-articular loose bodies. Follow-up contrast-enhanced MRI demonstrated progression of the lesion, which now measured approximately 3.9 × 5.7 cm and exhibited high T1 signal intensity suggestive of hemorrhagic content. There was clear communication between the lesion and the AC joint. A new marginally enhancing lytic lesion was also identified in the lateral clavicle, with differential diagnoses including active erosive changes and early osteomyelitis. Furthermore, extensive surrounding soft tissue and synovial enhancement were observed, raising concern for complicated inflammatory bursitis or a synovial neoplastic process (Figures 2, 3).

Surgical procedure

The patient underwent excision of the left shoulder mass under general anesthesia in the beach chair position. A 10-cm incision was made over the mass. Dissection revealed a connection between the lesion and the AC joint, and subperiosteal dissection was performed. The specimen was sent for histopathological examination and culture. Hemostasis was achieved, the wound was irrigated with normal saline, and layered closure was performed with skin staples. A sterile dressing was applied.

Histopathology report

**Figure 4:**
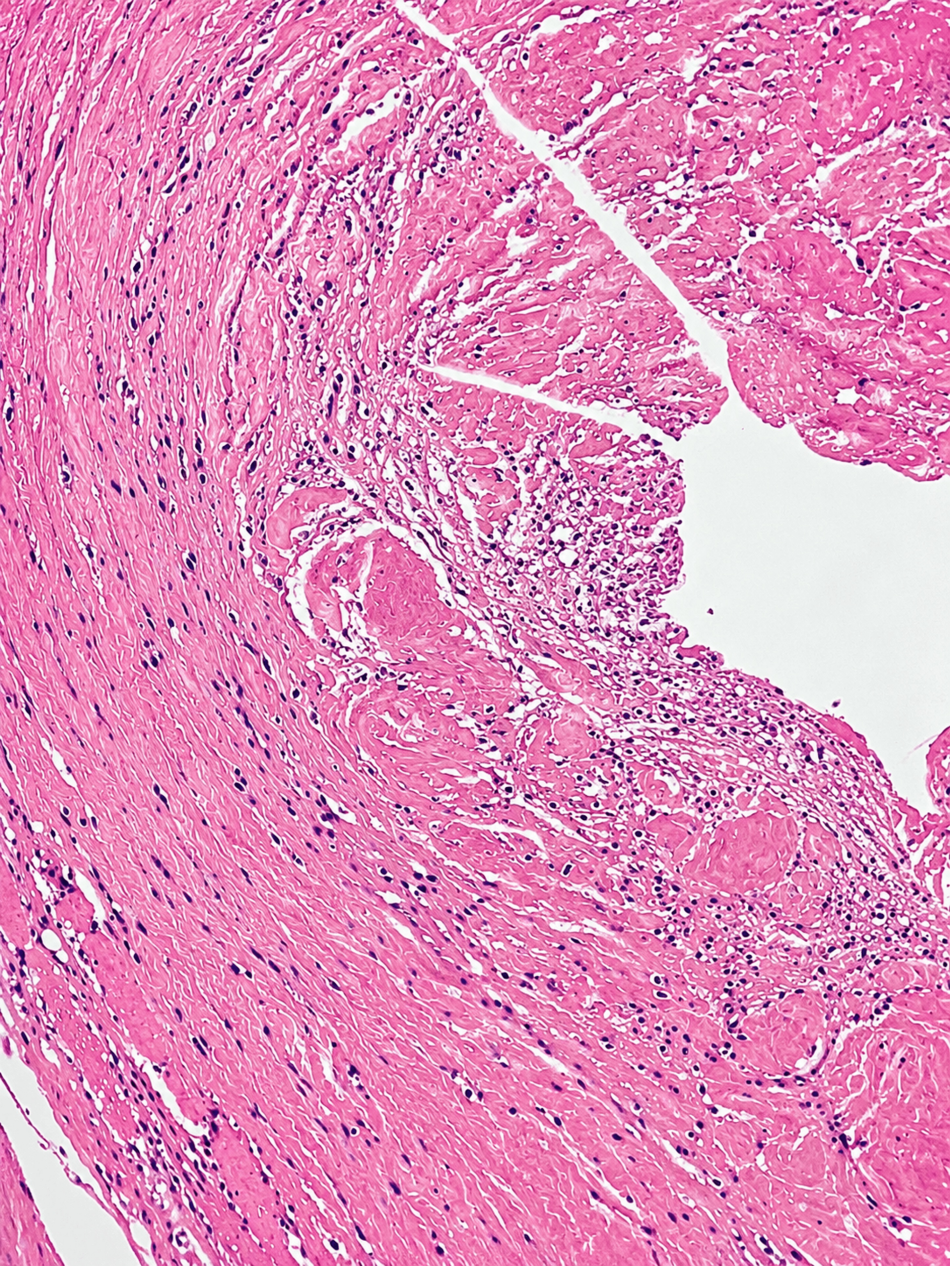
Histopathological section demonstrating a cystic cavity lined by dense fibrocollagenous tissue without a true epithelial lining, associated with eosinophilic degenerative material and focal chronic inflammatory changes, consistent with a pseudocyst.

**Figure 5:**
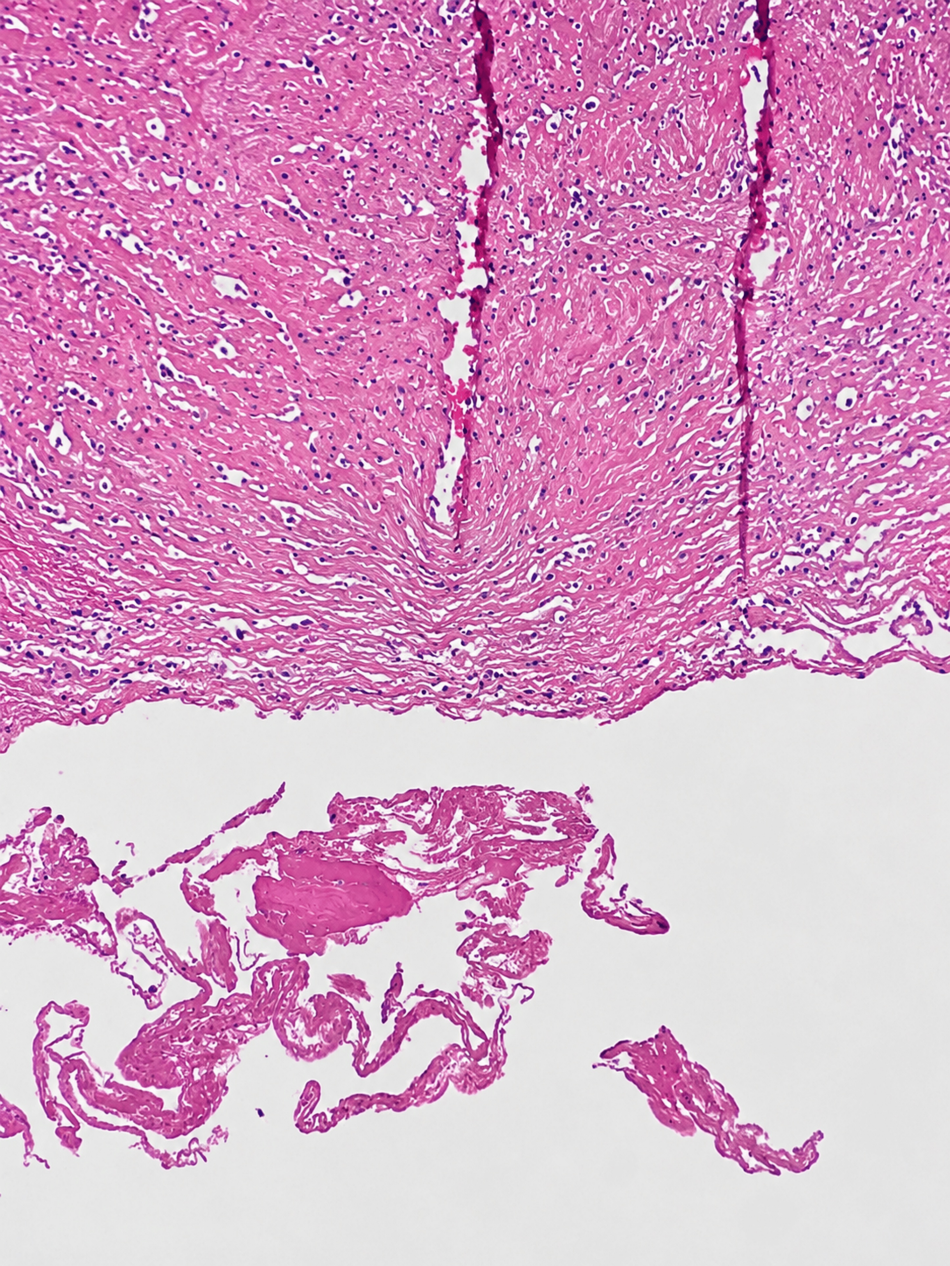
Histopathological section (H&E stain) providing a low-power view of the shoulder mass. The image demonstrates a cystic cavity lacking an epithelial lining, characteristic of a pseudocyst. The wall is composed of dense fibrocollagenous tissue with evidence of chronic inflammatory changes, consistent with a Geyser phenomenon-associated acromioclavicular joint cyst.

**Figure 6:**
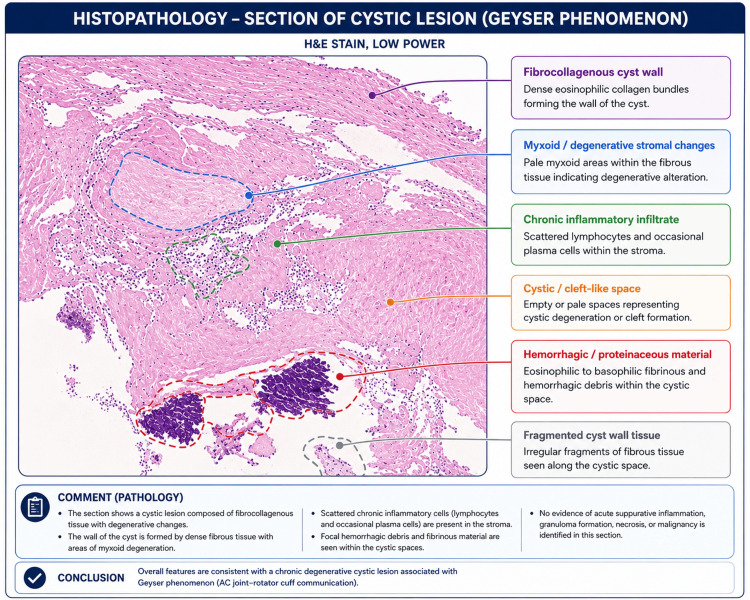
Histopathology of the excised acromioclavicular joint cyst. Note: Labels and annotations were created by the authors using Canva® (Canva Pty Ltd., Sydney, Australia) based on the original histopathology slide. No AI-generated images were used.

The specimen consisted of three pieces of the left distal clavicle and a left shoulder soft tissue mass obtained by lumpectomy, measuring 6 × 4 × 3 cm in aggregate. Gross examination of the mass revealed a well-circumscribed solid and cystic lesion measuring 3.5 × 2.5 × 2 cm, with surgical margins of 0.5 cm (distal), 0.4 cm (proximal), 0.3 cm (anterior), 0.3 cm (posterior), 0.5 cm (medial), 0.5 cm (lateral), and 0.4 cm (deep). Histopathological examination of the excised left shoulder mass and distal clavicle revealed a well-circumscribed cystic lesion lacking an epithelial lining, consistent with a benign inflammatory pseudocyst. The lesion contained fibrinoid, degenerated, and focally mucoid material and was surrounded by a thick fibrous capsule with mild acute-on-chronic inflammatory changes. The adjacent soft tissue showed benign fibroadipose tissue with areas of fat necrosis and a foreign body-type giant cell reaction. The distal clavicle specimen demonstrated benign bone spicules lined by fibrocollagenous tissue and fibrinoid material with mild inflammation. The lesion was completely excised, and no evidence of malignancy was identified. Overall, the findings were consistent with complicated bursitis or chronic degenerative shoulder pathology (Figures 4-6).

Follow-up

At the three-month follow-up, the patient demonstrated complete resolution of the clinical signs and symptoms related to the AC joint cyst. He reported complete satisfaction with the surgical outcome. Physical examination revealed a well-healed surgical incision, no residual swelling or palpable mass, preserved full range of motion of the left shoulder, and no neurovascular deficits. The patient expressed no cosmetic concerns regarding the surgical scar. There was no evidence of cyst recurrence or postoperative complications at follow-up.

## Discussion

There is limited literature on AC joint cysts and the Geyser phenomenon. AC joint cysts usually present as a painless mass over the AC joint and are most commonly seen in elderly patients. Although a few reports have described these cysts in the absence of rotator cuff pathology [4], most studies have demonstrated a strong association with massive rotator cuff tears [1].

The development of AC joint cysts is thought to result from chronic degenerative changes in the shoulder. Massive rotator cuff tears can lead to superior migration of the humeral head and degeneration of both the glenohumeral and AC joints. Over time, this may create communication between the joints, allowing synovial fluid to track superiorly and form a cyst. In our patient, the large cyst, prolonged clinical history, and associated full-thickness rotator cuff tear support this mechanism. Similar cases reported in the literature have primarily involved elderly patients with advanced rotator cuff disease.

The Geyser phenomenon is not merely an imaging finding but also an indicator of significant underlying shoulder pathology. It typically occurs in the setting of chronic full-thickness rotator cuff tears with advanced degenerative changes of the shoulder. Leakage of synovial fluid through the rotator cuff defect into the AC joint reflects longstanding disruption of normal shoulder anatomy. Therefore, the presence of the Geyser sign should prompt evaluation for cuff tear arthropathy and advanced rotator cuff insufficiency. Communication between the glenohumeral and AC joints remains the hallmark of the Geyser phenomenon. When Craig first described AC joint cysts in 1986 [5], arthrography was used to demonstrate the escape of contrast through the AC joint, producing the characteristic "Geyser sign." Today, MRI is the preferred imaging modality because it can clearly demonstrate the cyst, associated rotator cuff pathology, and communication between the joints.

In our case, the patient presented with a gradually enlarging shoulder mass that initially raised concern for malignancy because of its size, progressive enlargement, advanced age, and family history of cancer. Soft tissue sarcoma was considered in the differential diagnosis; however, MRI demonstrated communication with the AC joint, and histopathological examination confirmed a benign lesion. An aneurysm was also considered because of the hemorrhagic appearance observed on the follow-up MRI, but the absence of pulsatility on examination and intraoperative findings excluded a vascular cause. Early osteomyelitis was considered because of the lytic clavicular lesion and contrast enhancement; however, the absence of systemic signs of infection and benign histopathological findings made this diagnosis unlikely. Synovial chondromatosis was another differential diagnosis because of the low-signal intra-articular foci observed on MRI, but no cartilaginous bodies were identified on histopathological examination.

The patient was treated with cyst excision and distal clavicle resection and had an excellent postoperative outcome. Most reported cases in the literature have also been managed surgically, particularly in the presence of progressive enlargement, diagnostic uncertainty, cosmetic concerns, or associated symptoms [6,7]. Aspiration is generally discouraged because recurrence is common, and fistula formation may occur [8]. Although short-term postoperative outcomes appear favorable, there is limited evidence regarding medium- and long-term follow-up. Further studies are needed to better define recurrence rates, functional outcomes, and the optimal management of AC joint cysts associated with the Geyser phenomenon.

## Conclusions

The Geyser phenomenon is a benign but uncommon manifestation of advanced rotator cuff pathology and glenohumeral degenerative disease. Although the presentation of a large, progressively enlarging shoulder mass may raise concern for malignancy, particularly in patients with a relevant family history, recognition of the characteristic communication between the cyst and the acromioclavicular joint on MRI can facilitate an accurate diagnosis and help avoid unnecessary oncologic investigations or overly aggressive interventions. In the present case, surgical excision combined with distal clavicle resection provided both a definitive histopathological diagnosis and an excellent clinical outcome, with no evidence of recurrence at follow-up. Increased awareness of this entity among clinicians is essential to ensure appropriate evaluation and management. Further studies with medium- and long-term follow-up are warranted to better characterize recurrence rates, functional outcomes, and the optimal management of acromioclavicular joint cysts associated with the geyser phenomenon.
